# Supplementary material for: 68Ga-PSMA PET/CT in Recurrent Prostate Cancer after Radical Prostatectomy Using PSMA-RADS Version 2.0
Source: Diagnostics (Basel). 2024 Jun 19;14(12):1291. doi: 10.3390/diagnostics14121291 (PMC11202891; doi:10.3390/diagnostics14121291)
Supplement: Supplementary file 1 [file diagnostics-14-01291-s001.zip › diagnostics-3010273-supplementary.pdf]

Table S1. PSMA-RADS scores (version 2.0) [7].

PSMA-RADS Score	Description
PSMA-RADS 1 (benign)	**Benign lesion** characterized by biopsy or pathognomonic finding on anatomic imaging and with focal radiotracer uptake
PSMA-RADS 2 (likely benign)	**Equivocal** (focal, but low level such as blood pool) **uptake in soft-tissue site atypical of PCa involvement** (e.g., axillary or hilar lymph nodes)**; equivocal uptake in bone lesion atypical of PCa involvement** (e.g., uptake fused to bone lesion and strongly suspected of being degenerative or another benign etiology). Upon follow-up, stable lesions without treatment are likely benign and could then be scored with PSMA-RADS 1 or 2
PSMA-RADS 3 (equivocal)	
PSMA-RADS 3A	**Equivocal uptake in soft-tissue site typical of PCa involvement** (e.g., pelvic or retroperitoneal lymph nodes). If targetable, biopsy may help confirm diagnosis. Alternatively, follow-up imaging (either anatomic or PSMA-targeted PET/CT) showing progression can establish diagnosis. We recommend an initial follow-up period of 3–6 months in oligometastatic patients (>5 metastases); it is at the reader's discretion to reclassify this lesion to PSMA-RADS 4 [a]
PSMA-RADS 3B	**Equivocal uptake in bone lesion not definitive but also typical of PCa on anatomic imaging** (i.e., pure marrow-based lesion with little, if any, surrounding bony reaction, lytic or infiltrative lesion, or classic osteoblastic lesion). Na18F-PET/CT or bone biopsy may be considered. Alternatively, follow-up imaging (either anatomic or PSMA-targeted PET/CT) with evidence of progression may confirm diagnosis. In oligometastatic patients (>5 metastases), it is at the reader's discretion to reclassify this lesion to PSMA-RADS 4 [a]
PSMA-RADS 3C	**Intense uptake in site highly atypical of all but advanced stages of PCa**, which requires further workup. Biopsy to confirm diagnosis histologically is often preferred, although organ-specific follow-up imaging may be considered (e.g., liver-protocol MRI to evaluate possible primary hepatocellular carcinoma)
PSMA-RADS 3D	**Any lesion on CT that requires further workup but does not show any tracer uptake.** Biopsy to confirm diagnosis is often preferred, although organ-specific follow-up imaging may be applicable
PSMA-RADS 4 (PCa highly likely)	**Intense uptake in site typical of PCa but lacking definitive findings on conventional imaging**[b]
PSMA-RADS 5 (PCa almost certainly present)	**Intense uptake in site typical of PCa and having corresponding findings on conventional imaging** [b]**,** although obtaining tissue for genomic analysis or other purposes may be useful
PSMA-RADS 5T (treated PCa metastasis)	**Previously identified metastases after treatment (e.g., irradiated sclerotic bone lesions) with or without uptake**
Overall RADS score	**Defined by the highest PSMA-RADS score of any of the individual target lesions**

CT—computed tomography; PC—prostate cancer; PET—positron emission tomography; PSMA-RADS—Prostate-Specific Membrane Antigen Reporting and Data System; RADS—reporting and data system.

[a] Lesion-based classification may be omitted in patients with large-scale metastases.

[b] Given the high specificity of PSMA expression in prostate cancer cells [20] and highly accurate detection rates for selected radiotracers [21,22], it is unlikely that biopsy confirmation will be needed.
